# Trans-arterial positive ICG staining-guided laparoscopic liver watershed resection for hepatocellular carcinoma

**DOI:** 10.3389/fonc.2022.966626

**Published:** 2022-07-22

**Authors:** Xinye Qian, Wang Hu, Lu Gao, Jingyi Xu, Bo Wang, Jiyong Song, Shizhong Yang, Qian Lu, Lin Zhang, Jun Yan, Jiahong Dong

**Affiliations:** ^1^ Center of Hepatobiliary Pancreatic Disease, Beijing Tsinghua Changgung Hospital, School of Clinical Medicine, Tsinghua University, Beijing, China; ^2^ School of Clinical Medicine, Tsinghua University, Beijing, China; ^3^ The State Key Laboratory of Precision Measurement Technology and Instruments, Department of Precision Instrument, Tsinghua University, Beijing, China; ^4^ Beijing Jingzhen Medical Technology Ltd., Beijing, China; ^5^ Department of Hepatobiliary surgery, Xuzhou Central Hospital, Xuzhou, China; ^6^ Department of Hepatobiliary Surgery, The No.2 Hospital of Baoding, Baoding, China

**Keywords:** hepatocellular carcinoma, liver watershed, arterial ICG staining, laparoscopic surgery, minimal invasive

## Abstract

**Introduction:**

Anatomical liver resection is the optimal treatment for patients with resectable hepatocellular carcinoma (HCC). Laparoscopic Couinaud liver segment resection could be performed easily as liver segments could be stained by ultrasound-guided indocyanine green (ICG) injection into the corresponding segment portal vein. Several smaller liver anatomical units (liver watersheds) have been identified (such as S8v, S8d, S4a, and S4b). However, since portal veins of liver watersheds are too thin to be identified under ultrasound, the boundaries of these liver watersheds could not be stained intraoperatively, making laparoscopic resection of these liver watersheds demanding. Digital subtraction angiography (DSA) could identify arteries of liver watersheds with a diameter of less than 2 mm. Yet, its usage for liver watershed staining has not been explored so far.

**Purpose:**

The aim of this study is to explore the possibility of positive liver watershed staining *via* trans-arterial ICG injection under DSA examination for navigating laparoscopic watershed-oriented hepatic resection.

**Methods:**

We describe, in a step-by-step approach, the application of trans-arterial ICG injection to stain aimed liver watershed during laparoscopic anatomical hepatectomy. The efficiency and safety of the technique are illustrated and discussed in comparison with the laparoscopic anatomical liver resection *via* ultrasound-guided liver segment staining.

**Results:**

Eight of 10 HCC patients received successful trans-arterial liver watershed staining. The success rate of the trans-artery staining approach was 80%, higher than that of the ultrasound-guided portal vein staining approach (60%). Longer surgical duration was found in patients who underwent the trans-artery staining approach (305.3 ± 23.2 min vs. 268.4 ± 34.7 min in patients who underwent the ultrasound-guided portal vein staining approach, *p* = 0.004). No significant difference was found in major morbidity, reoperation rate, hospital stay duration, and 30-day and 90-day mortality between the 2 groups.

**Conclusions:**

Trans-arterial ICG staining is safe and feasible for staining the aimed liver watershed, navigating watershed-oriented hepatic resection under fluorescence laparoscopy for surgeons.

## Introduction

Surgery remains the most effective therapy for patients with resectable hepatocellular carcinoma (HCC) (1). Furthermore, anatomical resection of liver segment decreases the risk of HCC recurrence and surgical complications (2, 3). Yet, many HCC patients were also diagnosed with chronic liver diseases, like cirrhosis (4). These patients with limited liver function might suffer more from postoperative complications and prolonged hospital days if they received routine liver segmentectomy, like segment 8 resection, even if the future liver volume (FLV) is sufficient (5). Regional liver resection is mainly performed for these patients. However, regional resection would increase the recurrence rate of HCC among these patients (6). It has been proved that there are liver anatomical units smaller than the Couinaud liver segment, like sub-segment S8V, S8D, S4A, and S4B (7, 8). The connotation of an anatomical liver unit is the watershed of liver where a specific branch of hepatic artery, portal vein, and bile duct dominates the region according to the Glission system, like a Couinaud segment, sub-segment, or even smaller segment (9). If surgeons could perform laparoscopic liver resection based on the watershed of the HCC, the surgical trauma could decrease to the lowest extent without harming the oncological effect of hepatectomy for HCC patients.

However, because liver is a solid organ, it is difficult to determine the border of liver watershed, although with the development of fluorescence laparoscopy, laparoscopic Couinaud liver segment resection has become possible (10). Surgeons could identify the border of the Couinaud segments by ultrasound-guided indocyanine green (ICG) injection of the corresponding segment portal vein. However, liver watershed (or sub-segments) staining under laparoscopy is almost impossible due to the limitation of the ultrasound-guided portal vein puncture technique, under which it is difficult to puncture branches of the segment portal vein. This study has created a trans-arterial ICG staining under digital subtraction angiography (DSA) to visualize liver watersheds (or sub-segments) so as to navigate laparoscopic watershed-oriented hepatic resection, which minimized surgical trauma for HCC patients while ensuring the oncological treatment effect.

## Method

A cohort study was conducted between September 2021 and February 2022 at Beijing Tsinghua Changgung Hospital. This study was approved by the Ethics Committee of Beijing Tsinghua Changgung Hospital under the Declaration of Helsinki and registered at http://www.chictr.org.cn/index.aspx (registration number ChiCTR2100054461). All patients included in this study provided written informed consent at Beijing Tsinghua Changgung Hospital.

### Patients

**Figure 1:**
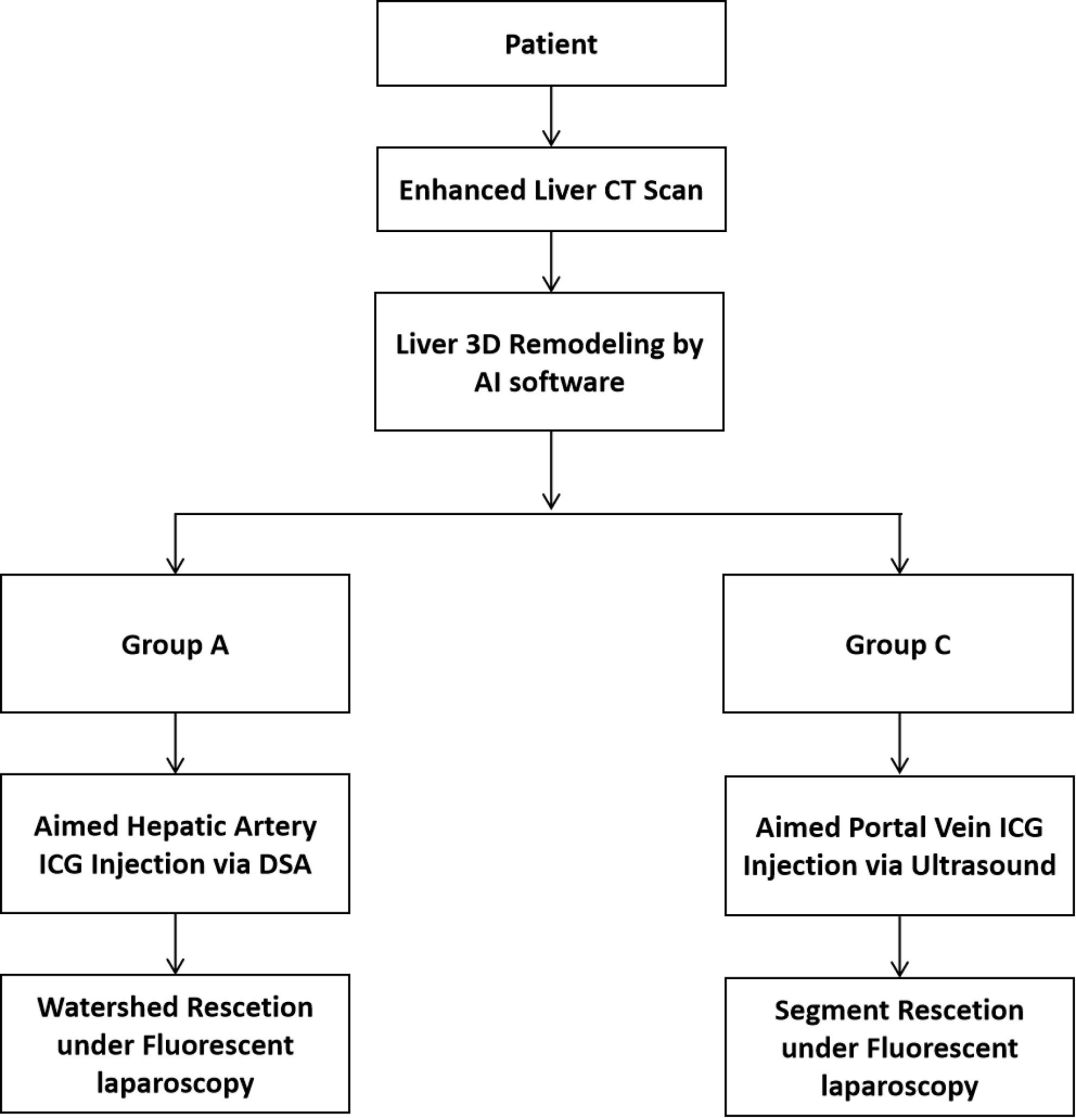
Flowchart of patient selection.

Patients for the trans-arterial positive ICG staining (Group A) were eligible for inclusion if (1) they were diagnosed with liver tumor (2); the longest diameter of the tumor was shorter than 4 cm (3); patients have Child–Pugh Grade A (4); patients have ASA Grade I–II (5); patients have no contraindication of laparoscopic surgery (6); patients have no contraindication of DSA operation; and (7) patients are not allergic to ICG. A control cohort (Group C) was composed of 68 patients who underwent fluorescent laparoscopic hepatectomy for pathologically confirmed HCC from January 2018 to February 2022. The exclusion criteria for Group C were as follows: (1) non-anatomical liver resection; (2) ICG-free laparoscopic surgery; (3) open conversion of the surgery; and (4) previous history of abdominal operation. A total of 10 and 20 HCC patients were included in Group A and Group C (**Figure 1**).

### Protocol

The pre-operative procedures for patient in both Groups A and C were similar. Patients received an enhanced CT scan. AI-based 3D liver remodeling software (Jingzhen Technology) was used to build a 3D liver model automatically (11), based on which the surgical plan was made. Surgical plans for patients in Group A was liver watershed resection according to the tumor location (for example, patients with tumor in S8v received a S8v resection), while surgical plans for patients in Group C received Couinaud segment resection according to the tumor location (for example, patients with rumor in S8v received an S8 resection).

All the patients received liver resection with the margin wider than 10 mm. However, the surgical protocol for the 2 groups is different. Patients in Group C underwent routine laparoscopic liver segmentectomy. Ten milliliters of ICG (diluted to 0.0125 mg/ml) was prepared. After abdominal entry, surgeons used an ultrasound-guided technique to puncture the aimed portal vein (portal veins of the Couinaud segments) and inject ICG at the speed of 1 ml/s for positive liver segment staining. After the aimed Couinaud segment was observed on fluorescence laparoscopy, the pringle maneuver was applied and the surgery was performed under fluorescence laparoscopy.

**Figure 2:**
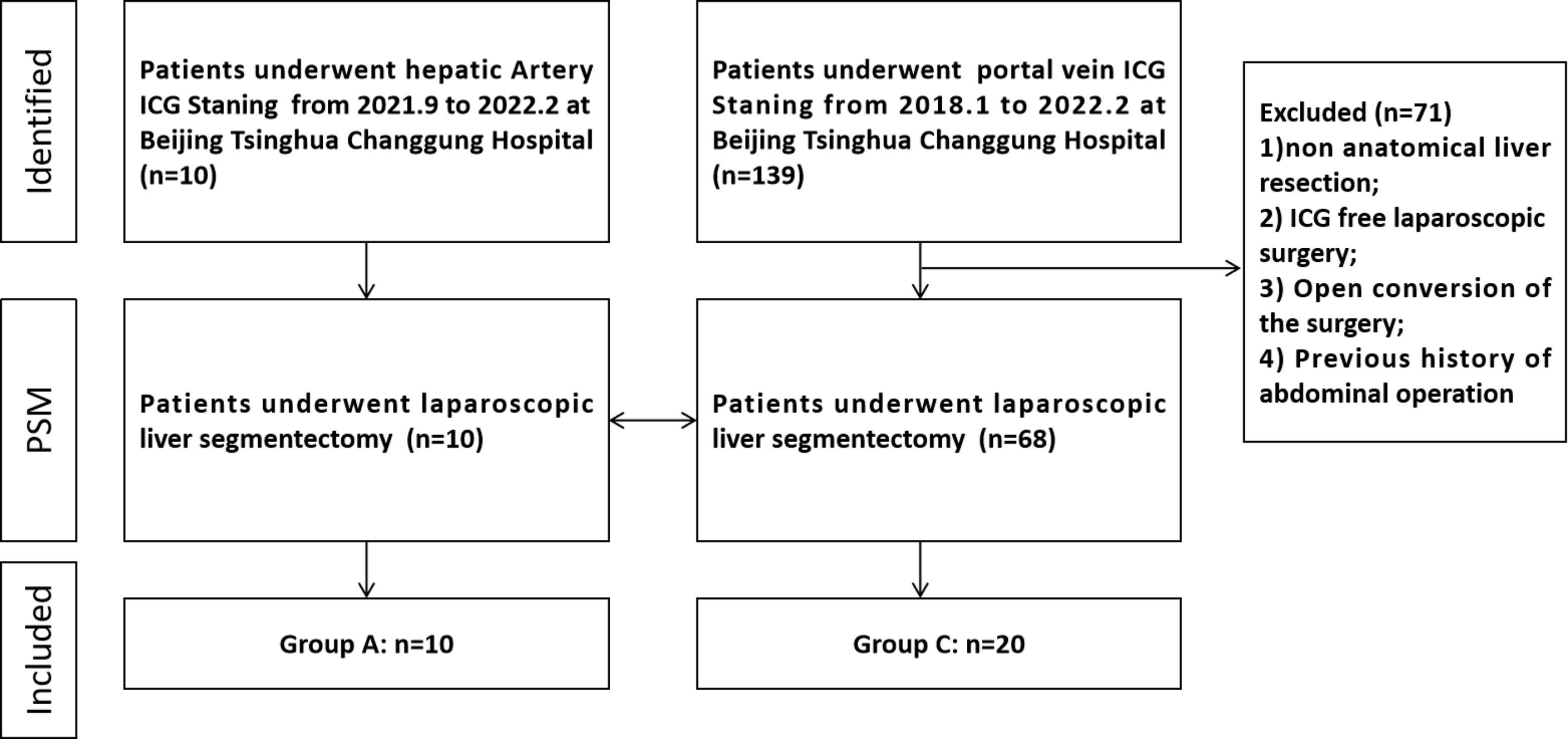
Protocol for patients who underwent liver watershed or segment resection.

After entering the hybrid operation room, patients in Group A received a femoral artery catheterization after anesthesia. After abdominal entry, the guide wire (DSA examination) entered the aimed hepatic artery (arteries of the liver watersheds). After confirmation, the far end of the selected artery, which was supplying the tumor, was embolized with gelatin sponge first. Then, ICG (diluted to 0.0125 mg/ml) was injected at the speed of 1 ml/s into the targeted artery. When the aimed liver watershed (sub-segment) was observed on fluorescence laparoscopy, the artery was embolized with gelatin sponge. Finally, the surgery was performed under fluorescence laparoscopy according to the staining border (**Figure 2**).

### Data collection

Demographic, anesthetic, and surgical information of all patients was documented to detect any statistical difference between the 2 groups. The definition of a successful liver watershed, sub-segment, or segment staining is that a clear border of the aimed area could be observed under fluorescence laparoscopy. One surgeon of the Hepatobiliary Pancreatic department, who was blinded to the trial, was assigned to evaluate whether the staining is successful. Post-hepatectomy liver failure (PHLF) was assessed according to the criteria established by the International Study Group of Liver Surgery (ISGLS); post-operative complications were classified using the Clavien–Dindo classification.

### PSM analysis

PSM analysis was employed to reduce bias in patient selection to investigate the differences between Group A and Group C. 1:2 PSM was performed based on the “nearest neighbor” method with a caliper width of 0.02. Variables including age, gender, α-fetoprotein, hemoglobin, total bilirubin, albumin, alanine aminotransferase (ALT), aspartate aminotransferase, gamma-glutamyl transferase, prothrombin time, international normalized ratio, HBsAg, HCV-RNA, Child–Pugh score, ASA grade, the number of tumors and maximal tumor size, and tumor location were comprehensively included in the calculation of the propensity score. All the matched indicators were measurements before surgery.

### Statistics

Statistical analyses were performed using SPSS software version 25.0 (IBM Corp., Armonk, NY), R software version 4.0.2 (R Foundation for Statistical Computing, Vienna, Austria), and GraphPad Prism 8 (LLC, San Diego, CA). Continuous variables were presented as medians (SD). Mann–Whitney *U*-test (Wilcoxon rank-sum test), Student’s *t*-test, Pearson *χ* ^2^ test, and Fisher’s exact test were used to assess statistical significance as appropriate. A repeated measures analysis of variance (ANOVA) was used for indicator analysis of multiple measurements postoperatively. *p* < 0.05 was considered statistical significance in the study.

## Results

### Patient characteristics

A total of 10 HCC patients who underwent hepatic artery staining (Group A) and a total of 20 HCC patients who underwent portal vein staining (Group C) were included in the study. The patients’ characteristics are shown in **Table 1**. There was no significant difference between two groups in demographic information, including age, sex, pathologic diagnosis, BCLC stage, MVI, ASA Grade, Child Pugh Grade. All patients had a single HCC located in one of the following segments: S3, S4b, S5, S8v, and S8d. Patients in Group A received liver watershed resection according to the tumor location (for example, patients with tumor in S8v received an S8v resection), while patients in Group C received Couinaud segment resection according to the tumor location (for example, patients with rumor in S8v received an S8 resection).

**Table 1:** Basic characteristics of HCC patients in Group A and Group C after PSM.

Characteristic	Group A	Group C	*p*
	*n* = 10	*n* = 20	
Age (years)	56.8 ± 6.3	55.25 ± 6.9	0.56
Sex (Male/Female)	8/2	17/3	>0.99
Pathologic Diagnosis			
Hepatocellular Carcinoma	10	20	>0.99
Others	0	0	>0.99
MVI (0/1/2)	7/3/0	12/7/1	>0.99
BCLC Stage (A1/A2/A3)	10/0/0	20/0/0	>0.99
HBV Infection (Positive/Negative)	10/0	20/0	>0.99
Tumor Location (Liver Segment)			
S8v	4	8	>0.99
S8d	1	2	>0.99
S3	3	6	>0.99
S4b	1	2	>0.99
S5	1	2	>0.99
Longest Diameter of the Tumor (CM)	3.13 ± 0.5	3.1 ± 0.6	0.89
ASA Grade (I/II)	9/1	17/3	>0.99
Comorbidities			
Hypertension	1	2	>0.99
Diabetes	0	1	>0.99
Varicose Vein	0	1	>0.99
Child Pugh Grade (A/B)	10/0	20/0	>0.99
Open Conversion (Yes/No)	0/10	0/20	>0.99

MVI, microvascular invasion; BCLC stage, Barcelona Clinic Liver Cancer Stage; ASA Grade, American Society of Anesthesiologists physical status; values are presented as n (%). Fisher’s exact and Student’s t-tests were used, as appropriate; *p < 0.05.

### Intraoperative findings

**Figure 3:**
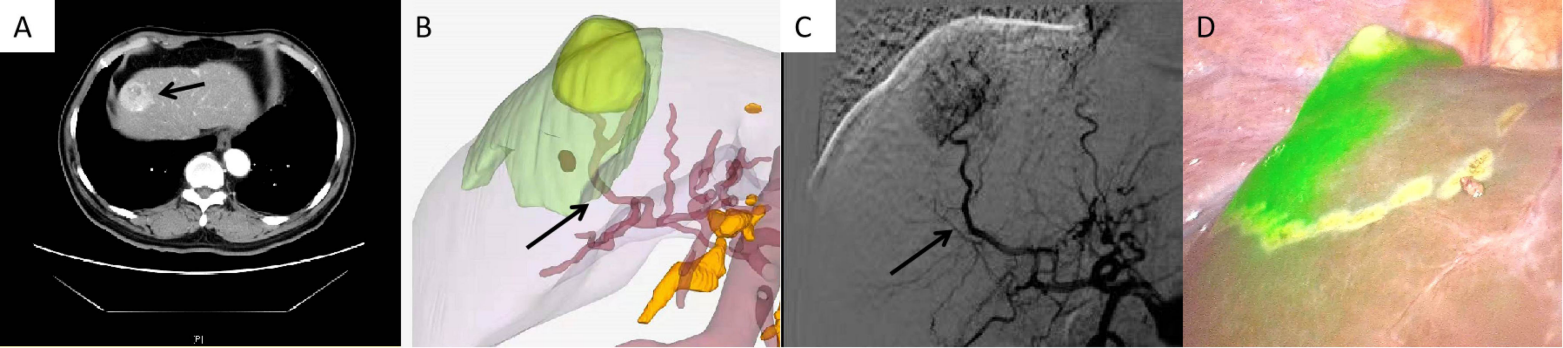
Case 1 (patent with HCC in S8v). **(A)** CT scan of HCC in S8v. **(B)** 3D modeling of the HCC and its watershed region. **(C)** Selected hepatic artery under DSA examination. **(D)** Positive ICG staining of S8v.

**Figure 4:**
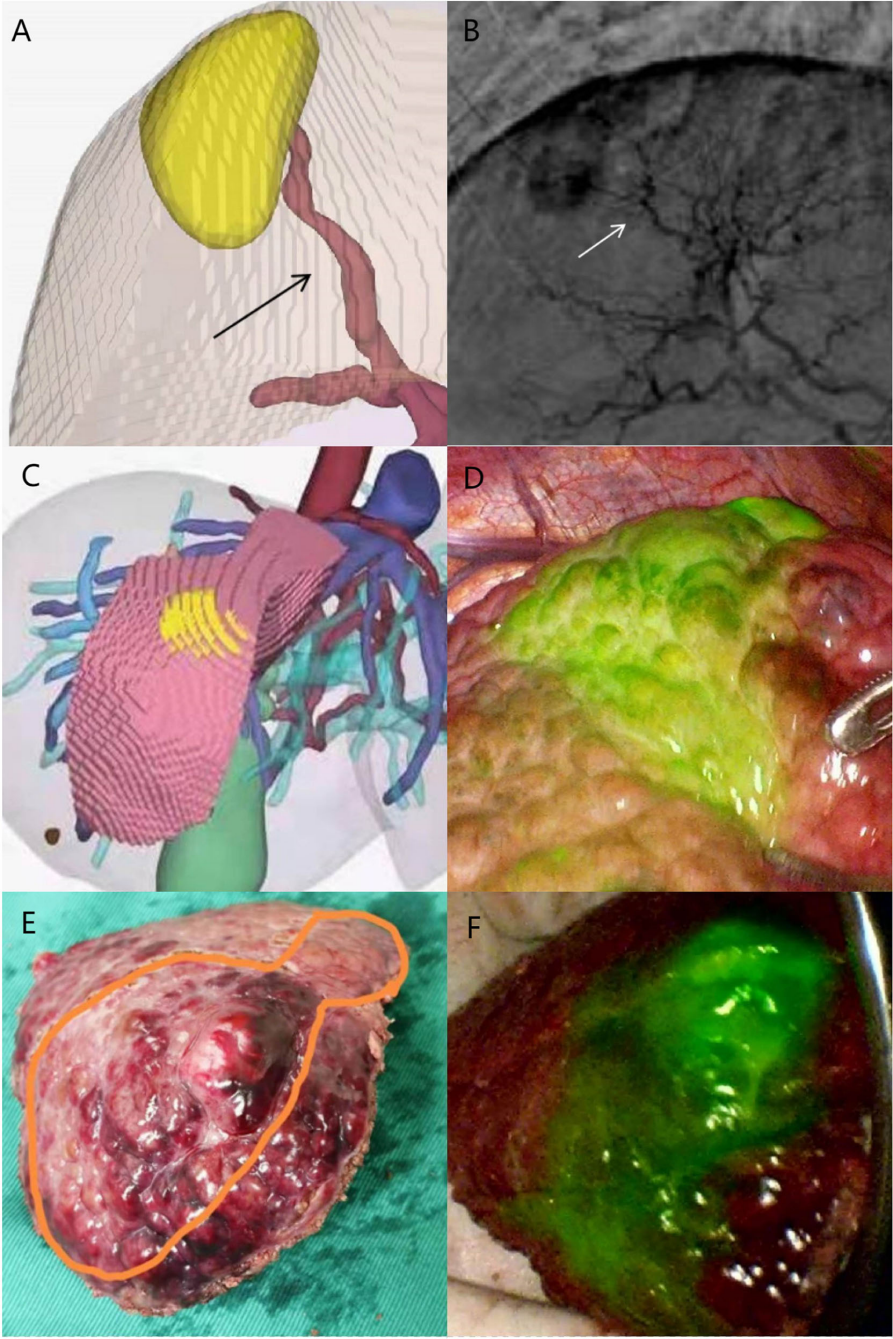
Case 2 (patent with HCC in S8v). **(A)** Selected hepatic artery on 3D modeling. **(B)** Selected hepatic artery under DSA examination. **(C)** 3D modeling of the HCC and its watershed region. **(D)** Positive ICG staining of S8v. **(E)** Surgical specimen. **(F)** Surgical specimen under fluorescence laparoscopy.

**Figure 5:**
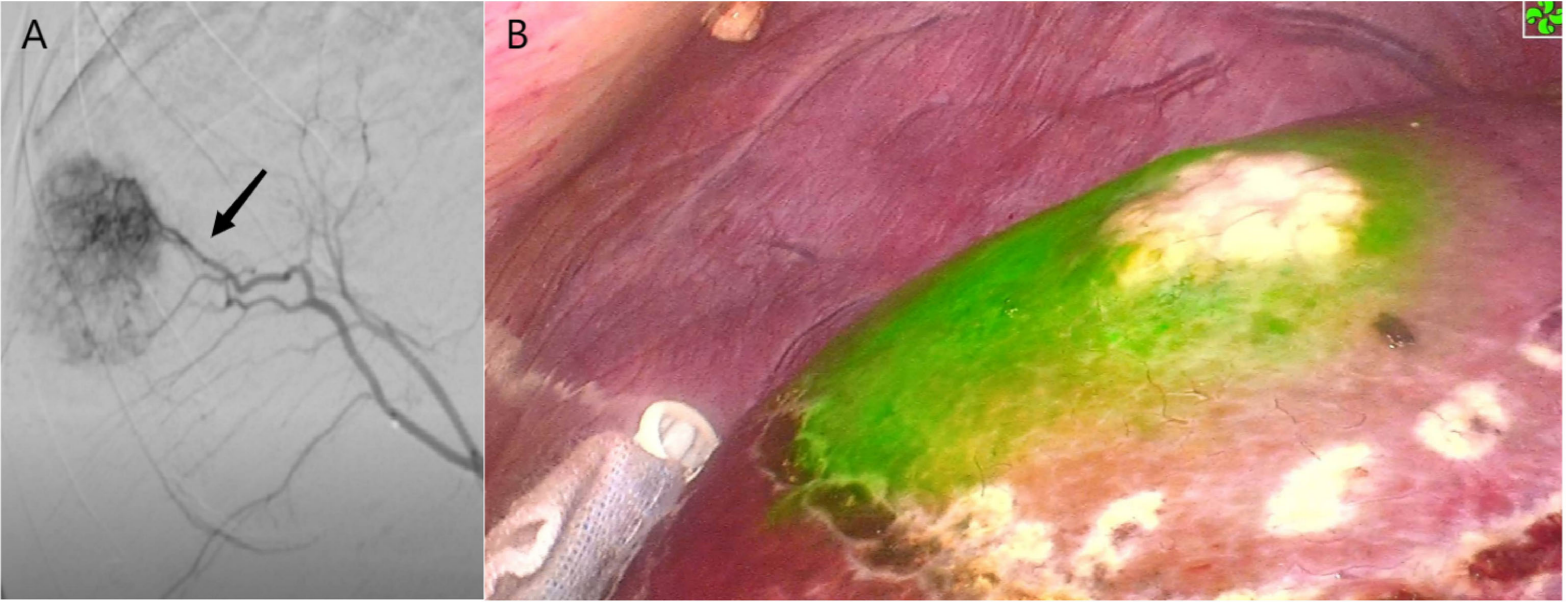
Case 5 (patent with HCC in S8d). **(A)** Selected hepatic artery under DSA examination. **(B)** Positive ICG staining of S8d.

**Figure 6:**
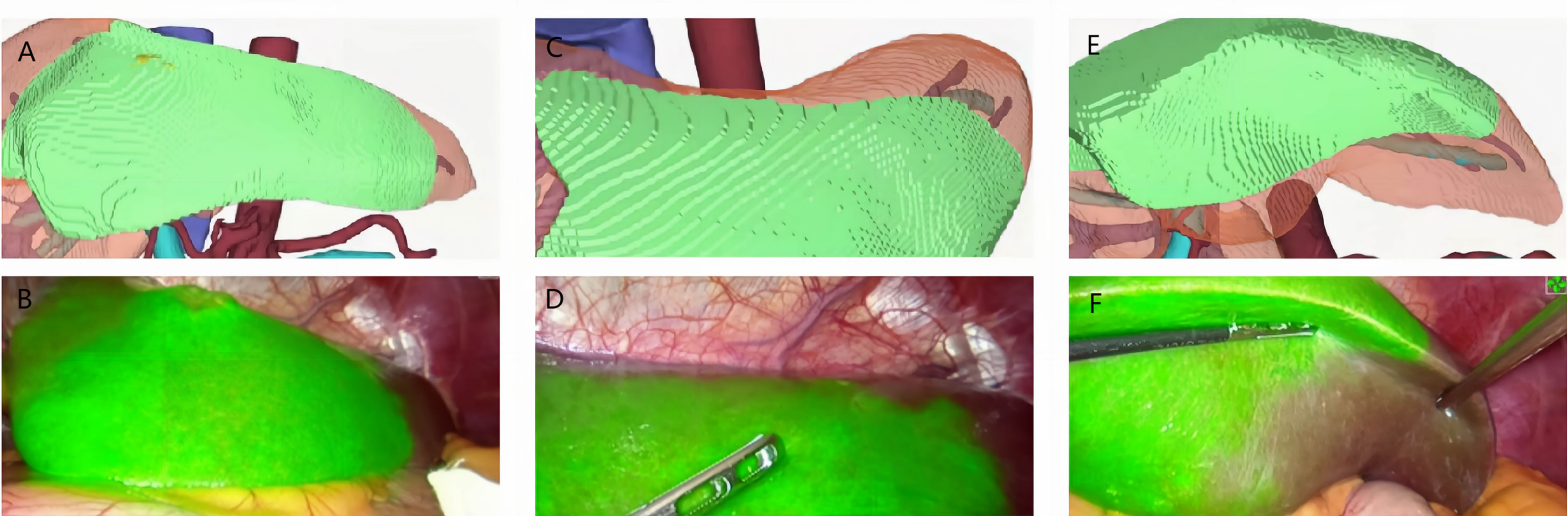
Case 7 (patent with HCC in S3). **(A, C, E)** 3D modeling of the HCC and its watershed region (S3). **(B, D, F)** Positive ICG staining of.

Eight out of 10 HCC patients in Group A received successful positive ICG staining of the aimed liver watershed, including S8v, S8d, and S3 (**Figures 3**–**6**). The staining of the 8 patients lasted until the end of the surgery. Twelve out of 20 HCC patients in Group C received successful segment staining, including S3, S4, S8, and S8, *via* portal vein puncture. Yet, no sub-segment (or watershed) staining was achieved in Group C. The overall staining success rate in Group A was 80%, while the success rate in Group C was 60%; however, no statistical difference was found (*p* > 0.05) (**Table 2**). Furthermore, in the 8 patients who received successful arterial staining in Group A, anatomical structures indicating the liver watershed borders were identified at the staining margin. Also, anatomical structures for the Couinaud segments were observed at the staining margin in the 12 patients who received successful conventional staining in Group C. The duration of the surgery was 305.3 ± 23.2 min in Group A, which was significantly longer than the 268.4 ± 34.7 min in Group C (*p* = 0.004) (**Table 2**).

**Table 2:** Surgical outcome in Group A and Group C.

Outcomes	Group A	Group C	*p*
	*n* = 10	*n* = 20	
Staining success rate (%, Yes/No)			
Overall	80% (8/2)	60% (12/8)	0.42
S8	100% (5/0)	60% (6/4)	0.23
S8v	100% (4/0)		
S8d	100% (1/0)		
S4	0% (0/1)	50% (1/1)	>0.99
S4b	0% (0/1)		
S3	100% (3/0)	67% (4/2)	>0.99
S5	0% (0/1)	50% (1/1)	>0.99
Surgery duration (min)	305.3 ± 23.2	268.4 ± 34.7	0.004*
Intraoperative bleeding (ML)	224.5 ± 70.6	202 ± 85.9	0.47
Pringle maneuver applied (Yes/No)	10/0	20/0	>0.99
Major morbidity (Clavien-Dindo grade >2)	0	2	>0.99
Reoperation	0	0	>0.99
Hospital stay duration (days)	7.5 ± 0.8	7.4 ± 0.9	0.66
30-day Mortality (Yes/No)	0/10	0/20	>0.99
90-day Mortality(Yes/No)	0/10	1/20	>0.99

Values are presented as n (%). Fisher’s exact and Student’s t-tests were used, as appropriate; *p < 0.05.

### Outcome

We next examined postoperative outcomes (**Table 2**) and found no significant difference in the average postoperative hospital stay for HCC patients in Group A and Group B (7.5 vs. 7.4 days, *p* > 0.05). Although no statistical difference was found in major morbidity between the 2 groups according to the Clavien–Dindo criteria, 2 of 20 patients in Group C underwent ultrasound-guided puncture and catheterization due to effusion at the surgical site or thoracic cavity (*p* > 0.05). The 90-day mortality rate for Group C was 5.0% (1/20); the patient died 2 months after his hepatectomy, most likely from pulmonary embolism as the patient suffered from diabetes and varicose vein before the surgery; also, ultrasound confirmed the existence of lower extremity deep venous thrombosis when the patient arrived at the emergency room 2 months after the surgery. Meanwhile, no deaths were reported within 90 days of hepatectomy in Group A. Yet, the difference in the 90-day mortality rates for the 2 study groups was not significant (*p* > 0.05).

## Discussion

Our results showed that trans-arterial ICG injection could stain liver watershed like Couinaud segments or sub-segments to assist surgeons performing complete laparoscopic watershed-oriented hepatic resection. Also, the technique showed great repeatability.

The concept of precision in liver surgery was proposed in 2013, in which the 3 pillars of precision liver surgery was safety, efficiency, and minimal invasion (12). That means an HCC patient should receive hepatectomy with the maximum surgical treatment effect and the minimum removed liver volume plus surgical invasion. Laparoscopic Couinaud segmentectomy is the most ideal therapy for patients with resectable HCC. However, many patients with HCC have limited liver function; for example, a Child–Pugh B patient might not survive resection of the liver segment 8. Troisi et al. have found that patients without preoperative portal hypertension and Child–Pugh B7 cirrhosis may benefit most from laparoscopic liver surgery, while the most of the remaining Child–Pugh B patients had a poor outcome because of surgical complications rather than tumor prognosis (13).

Fortunately, several liver sub-segments (S4A, S8V, S8D, etc.) have been identified. Also, the concept of liver watershed (liver anatomic unit) was proposed as the liver region dominated by the same branch of hepatic artery, portal vein, and bile duct (14). Various approaches have been tried to realize liver sub-segment (or liver watershed) resection. These approaches mainly include counterstaining techniques (injection of ICG through peripheral vein after separation and ligation of the target hepatic pedicle, or compression of the target portal vein in open surgery) (15, 16). Although laparoscopic S8d guided by counterstaining has been achieved by the Spanish scholars, the delicate skills required for the separation of the target hepatic pedicle under laparoscopy make it difficult to repeat in other patients or medical centers (17). In addition, dye invasion into the surgical targeted area is common in counterstaining technique, which could eventually lead to the failure in border identification. Moreover, the separation of the hepatic pedicle of the sub-segment could sometimes be impossible and dangerous, which might result in extra bleeding or other surgical complications.

As a branch of hepatic artery, portal vein, and bile duct dominates a certain liver watershed according to the Glisson system (9), hepatic artery staining, as well as portal vein staining, should represent the liver watershed, which was proved by a pre-clinical study on pigs using trans-arterial staining (18). Meanwhile, DSA has been proven to be both efficient and safe for intravascular therapy, including extremely small intracranial aneurysm of less than 2 mm (19). With the guidance of DSA examination, Grade 4 (or higher) hepatic arteries could be reached; thus, the liver watershed (or sub-segment) staining could be achieved *via* injection of the ICG through the aimed hepatic artery. Li et al. have proved that intra-arterial ICG injection provided a real-time image-guided intraoperative demarcation of targeted liver Couinaud segments (20). However, the liver watershed (or sub-segment) staining *via* the hepatic artery has not been explored in that study.

Our result showed that positive liver watershed (or sub-segment) staining *via* selected arterial ICG injection could be realized and repeatable, with an 80% success rate, higher than that of Group C (60%), although no statistical difference was observed (*p* > 0.05). Also, the anatomical structures of the liver watersheds were found at the margin of the staining area, indicating that the staining area was consistent with that of the portal vein approach. Possible reasons for this are as follows: (1) It is easier to identify vessels by DSA examination as contrast agent could be used during the process (21). (2) Surgeons can directly observe the location of the tumor during DSA examination, which makes it much easier to find the corresponding artery of the liver watershed. (3) The flow direction is always forward in the artery while it might be turbulence in the portal vein (22). (4) Embolism of the targeted artery is certain under DSA.

The reason for 2 failure cases in the arterial staining group (one tumor located in S5; another tumor located in S4b) is that we did not block the far end of the selected liver watershed artery (the part of artery supplying the tumor). When ICG was injected, the majority of the blood flow might flush into the tumor instead of the whole liver watershed because of tumor steal syndrome (23). Thus, we improved our protocol to embolize the far end of the selected liver watershed artery. This approach has led us to the successful staining in the rest of the patients in Group A.

The surgical duration is 37 min longer in Group A than in Group C, which was caused by the DSA examination and embolism of the targeted artery. Yet, no difference was found in other surgical outcomes from both groups, including postoperative major morbidity, reoperation rate, hospital stay duration, and 30- and 90- day mortality, according to which we believed prolonged duration of an average of 37 min is fully acceptable for patients who underwent hepatectomy.

Based on the above evidence, we believed that this approach might also allow more surgeons to perform laparoscopic liver watershed or sub-segment resection as DSA examination has become a routine technique in many medical centers worldwide. Our study does have limitations. The mean flaw was the relatively small number of patients enrolled. With an increased number of participants, a statistically significant increase in staining success rate might be observed in HCC patients who underwent trans-arterial positive liver watershed staining. However, as all the techniques used in the study were proved and common in clinical settings, the results of the study could infer that it is feasible to stain liver watershed *via* selected hepatic arterial ICG injection to navigate laparoscopic liver watershed resection. Further studies could be focused more on the surgical and oncological outcomes of the procedure in HCC patients with limited liver function, like patients in Child–Pugh B stage. Also, the trans-arterial ICG staining method might promote multiple liver watershed staining (like staining the combination of S8v and S4b so that the patient could receive resection of S8v+S4b instead of resection of S8+S4) as every hepatic artery of the targeted watershed could be located, injected with ICG, and embolized. These results are worth looking forward to.

## Conclusion

The present study demonstrated that trans-arterial ICG staining is safe and feasible for positive liver watershed staining to navigate watershed-oriented hepatic resection under fluorescence laparoscopy to minimize surgical trauma without harming the therapeutic effect of anatomic liver resection.

## Data availability statement

The original contributions presented in the study are included in the article/**Supplementary Material**. Further inquiries can be directed to the corresponding authors.

## Ethics Statement

The studies involving human participants were reviewed and approved by the ethic committee of Beijing Tsinghua Changgung Hospital. The patients/participants provided their written informed consent to participate in this study.

Written informed consent was obtained from the individual(s) for the publication of any potentially identifiable images or data included in this article.

## Author Contributions

XQ and JY conceived the project. XQ, WH, LG, and JX analyzed the data, and wrote the paper. XQ, LZ, and JY contributed to the surgery. QL and JD provided expert guidance and suggestions. All authors contributed to the article and approved the submitted version.

## Funding

This work is supported by the Start-up Fund for Talent Researchers of Tsinghua University (No. 10001020507) and the National Nature Foundation of China (No. 82090050, No. 82090053, and No. 81372272).

## Conflict of Interest

Author BW was employed by Beijing Jingzhen Medical Technology Ltd.

The remaining authors declare that the research was conducted in the absence of any commercial or financial relationships that could be construed as a potential conflict of interest.

## Publisher’s Note

All claims expressed in this article are solely those of the authors and do not necessarily represent those of their affiliated organizations, or those of the publisher, the editors and the reviewers. Any product that may be evaluated in this article, or claim that may be made by its manufacturer, is not guaranteed or endorsed by the publisher.
